# Knowledge, attitudes and practices of healthcare workers during the early COVID-19 pandemic in a main, academic tertiary care centre in Saudi Arabia

**DOI:** 10.1017/S0950268820001958

**Published:** 2020-08-28

**Authors:** M. H. Temsah, A. N. Alhuzaimi, N. Alamro, A. Alrabiaah, F. Al-Sohime, K. Alhasan, J. A. Kari, I. Almaghlouth, F. Aljamaan, A. Al-Eyadhy, A. Jamal, M. Al Amri, M. Barry, S. Al-Subaie, A. M. Somily, F. Al-Zamil

**Affiliations:** 1 College of Medicine, King Saud University, Riyadh, Saudi Arabia; 2 Department of Pediatrics, King Saud University Medical City, Riyadh, Saudi Arabia; 3 Prince Abdullah Bin Khaled Coeliac Disease Chair, Faculty of Medicine, King Saud University, Saudi Arabia; 4 Cardiac Science Department, King Saud University Medical City, Riyadh, Saudi Arabia; 5 Department of Family and Community Medicine, King Saud University Medical City, Riyadh, Saudi Arabia; 6 Prince Sattam bin Abdulaziz Research Chair for Epidemiology and Public Health, King Saud University, Riyadh, Saudi Arabia; 7 Department of Pediatrics, King Abdulaziz University, Jeddah, Saudi Arabia; 8 College of Medicine Research Center, King Saud University, Riyadh, Saudi Arabia; 9 Adult Critical Care Department, King Saud University, King Saud University Medical City/King Khalid University Hospital, Riyadh, Saudi Arabia; 10 Department of Infectious Disease, King Faisal Specialist Hospital and Research Center, Riyadh, Kingdom of Saudi Arabia; 11 Infectious Disease Unit, Department of Internal Medicine, King Saud University, Riyadh, Saudi Arabia; 12 Department of Pathology and Laboratory Medicine, College of Medicine, King Saud University and King Saud University Medical City, Riyadh, Saudi Arabia

**Keywords:** COVID-19, flu, healthcare workers, KAP, MERS-CoV

## Abstract

As the Middle East respiratory syndrome coronavirus (MERS-CoV) continues to occur in small outbreaks in Saudi Arabia, we aimed to assess the knowledge, attitudes and intended practices of healthcare workers (HCWs) during the early stage of the COVID-19 pandemic and compare worry levels with previous findings during the MERS-CoV outbreak in 2015. We sent an adapted version of our previously published MERS-CoV questionnaire to the same cohort of HCWs at a tertiary hospital in Saudi Arabia. About 40% of our sample had previous experience with confirmed or suspected MERS-CoV patients, and those had a significantly higher knowledge score (13.16 ± 2.02 *vs.* 12.58 ± 2.27, *P* = 0.002) and higher adherence to protective hygienic practices (2.95 ± 0.80 *vs.* 2.74 ± 0.92, *P* = 0.003). The knowledge scores on COVID-19 were higher in the current cohort than the previous MERS-CoV outbreak cohort (68% *vs.* 79.7%, *P* < 0.001). HCWs from the current cohort who felt greater anxiety from COVID-19 compared to MERS-CoV were less likely to have been exposed to MERS-CoV infected/suspected cases (odds ratio (OR) = 0.646, *P* = 0.042) and were less likely to have attended the hospital awareness campaign on COVID-19 (OR = 0.654, *P* = 0.035). We concluded that previous experience with MERS-CoV was associated with increased knowledge and adherence to protective hygienic practices, and reduction of anxiety towards COVID-19.

## Introduction

Since the World Health Organization (WHO) declared coronavirus disease 2019 (COVID-19) as a pandemic, it has become a major challenging public health problem worldwide [1, 2]. This pandemic has affected all aspects of people's life in almost all nations and among all socioeconomic groups. Healthcare workers (HCWs) of all types are facing an unprecedented crisis with the rapid spread of COVID-19 and severity of the disease in many infected individuals. As such many healthcare systems have been overwhelmed and HCWs presented with depression and anxiety [3, 4]. There is a potential shortage of physical resources, such as ventilators and intensive care unit beds, needed to care for surges of critically ill patients [5]; however, additional medical supplies and beds will be of limited help unless there is an adequate medical workforce.

The mental impact of the COVID-19 epidemic on general population [6], psychiatric patients [7], workers [8] patients [9], children [10], older adults [11] and medical students [12] has been reported. However, little attention has been paid to the psychological wellbeing and fatigue levels among HCWs [13, 14].

To further understand the knowledge, attitudes and intended practices of HCWs during the early stage of the COVID-19 pandemic, it is particularly beneficial to obtain their input, especially in an area of the world where other respiratory viral illnesses are either endemic, such as MERS-CoV, or seasonal, such as influenza.

## Methods

This study was the baseline for a serial, cross-sectional survey among HCWs in a tertiary care hospital with a 1000-bed capacity in Riyadh, Saudi Arabia. The HCWs had multinational backgrounds; in addition to Saudi nationals, they were mostly from the Indian subcontinent and the Philippines. Data were collected between 5 and 16 February 2020, which was just before the presentation of the first case of COVID-19 in Saudi Arabia.

The survey was a pilot-validated, self-administered questionnaire that was sent to HCWs online. The questionnaire was similar to that used in our previous MERS-CoV study [15], with modification and additional questions related to the current COVID-19 pandemic. The questions queried the demographic characteristics of the respondents (job category, age, sex, work area and years of clinical experience) and previous exposure to MERS-CoV patients, either suspected or confirmed cases. We assessed the following domains for every participant: KAP scores [16], knowledge about COVID-19 [17], HCW attitudes toward infection control measures [18] and hygienic practice change scores.

In addition, we assessed [19] the perceived adequacy of COVID-19 information and [15] perceived high fear/stress from the COVID-19 pandemic as compared to the previous MERS-CoV outbreak.

The HCWs knowledge of COVID-19 disease was tested using eight TRUE/FALSE questions (Supplementary Table S1). The perceived adequacy of knowledge, hygienic practice changes and HCW attitudes toward infection control measures were assessed using a series of Likert-based questions (Supplementary Tables S2–S4). The practice score is measuring the degree of improvement in protective practices after COVID-19, from 1 to 4, with 1 representing no change, 2 little change, 3 moderate change and 4 significant change. The attitude score represents the level of agreement with protective HCWs' attitudes toward infection control. The score ranges from 1 to 5, with 1 representing ‘strongly disagreeing’ and 5 representing ‘strongly agreeing’ with attitudes.

Additionally, the sources of information on the outbreak and attendance at the COVID-19 awareness campaign (Educational Day conference) that was conducted at the hospital in the first week of February 2020 were evaluated. The participants were asked about their worry level regarding the current COVID-19 compared to their worry level from the MERS-CoV outbreaks.

### Statistical analysis

We analysed the data using SPSS IBM V20 (SPSS, Inc., Chicago, IL, USA). For all tests, statistical significance was set at *P* < 0.05. For the five domains we used a summative score to summarise the results from continuous Likert's scale-based questions for each participant. An unpaired *t* test and analysis of variance (ANOVA) analysis followed various post hoc tests to compare the means of different groups. The model was significant based on a model goodness-of-fit Hosmer–Lemeshow test [*χ*^2^_(8)_ = 7.4, *P* = 0.490, Model AUC ROC = 74%, *χ*^2^_(16)_ = 103.8, *P* < 0.001].

The Pearson's (*r*) test was used to assess the bivariate associations between the measured HCW scores. Fisher's exact tests were used to establish the differences between HCW groups (physicians *vs.* non-physicians) for nominal variables. We compared this current analysis with data from our previous study conducted during the MERS-CoV outbreak in 2015 in the same institution [15].

The study was approved by the Institutional Review Board at the College of Medicine and King Saud University Medical City (approval no. 20/0065/IRB).

## Results

The questionnaire was sent to 800 HCWs with a 72.8% response rate; 582 HCWs completed the questionnaire.

**Fig. 1.**
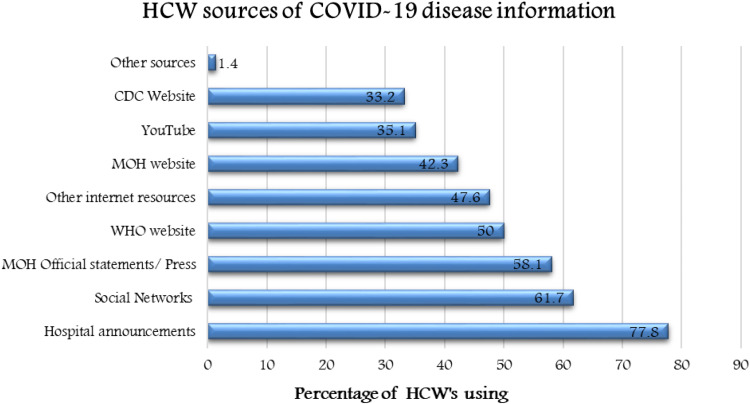
HCW sources of COVID-19 disease information.

The majority of participating HCWs were female (75%) and the most common age group was 31–39 years (38.3%, mean 38.6). Nurses constituted 62% of our study population. The majority of respondents were HCWs working in critical care units (44.8%) followed by outpatient clinics (28%), and inpatients wards (19.4%), see Table 1. HCWs used multiple sources of information about COVID-19, as shown in Figure 1.

**Table 1.** Descriptive statistics of the HCWs' demographics and bivariate analysis of the HCWs' measured knowledge, perceived adequacy of information, hygienic practice scores and attitudes toward hygiene considering statistically significant mean differences across demographic and professional characteristics and practices

	Frequency (%)	COVID-19 knowledge score (mean ± s.d.)	Perceived adequacy of COVID-19 information score (mean ± s.d.)	Hygienic practice score (mean ± s.d.)	HCW attitudes score (mean ± s.d.)
Whole sample		12.75 (2.2)	3.76 (0.98)	2.76 (0.91)	4.10 (1.02)
Maximum score range		0–16	1–5	1–4	1–5
Sex					
Male	145 (24.9)	12.80 (2.10)	3.58 (1.03)	2.36 (0.90)	3.85 (0.93)
Female	437 (75.1)	12.82 (2.24)	3.88 (0.93)	2.98 (0.82)	4.15 (1.10)
*t* test unpaired		*t* = 0.10, df = 615, *P* = 0.717	*t* = 3.19, df = 227.3, *P* = 0.002	*t* = 7.7, df = 580, *P* < 0.001	*t* = 3.03, df = 580, *P* = 0.003
Age groups	Mean age = 38.6, s.d. = 11.6 years				
≤30 years	178 (30.6)	12.64 (2.16)	3.64 (0.96)	2.55 (0.92)	3.9761 (1.02)
31–39 years	223 (38.3)	12.92 (1.92)	3.81 (1.04)	2.95 (0.85)	4.0953 (1.20)
40–49 years	133 (22.9)	12.80 (2.60)	3.96 (0.87)	2.98 (0.82)	4.1635 (0.84)
⩾50 years	48 (8.2)	13.0 (2.26)	4.02 (0.73)	2.87 (0.85)	4.125 (0.94)
One-way ANOVA		*F*(3,578) = 0.68, *P* = 0.567	*F*(3,199.8) = 4.21, *P* = 0.006	*F*(3,578) = 6.77, *P* < 0.001	*F*(3,578) = 0.93, *P* = 0.423
Clinical role					
Senior Physician	56 (9.6)	12.89 (1.61)	3.65 (0.94)	2.05 (0.76)	3.94 (0.81)
Registrar	52 (8.9)	12.54 (1.38)	3.57 (0.92)	2.40 (0.79)	3.89 (0.85)
Resident	48 (8.2)	12.29 (2.10)	3.42 (0.96)	2.22 (0.84)	3.78 (0.85)
Nurse	363 (62.4)	13.00 (2.21)	4.01 (0.90)	3.20 (0.68)	4.19 (1.11)
Auxiliary services	29 (5)	12.28 (3.95)	3.50 (1.10)	2.67 (0.99)	4.04 (1.03)
Intern	34 (5.8)	12.29 (1.64)	3.10 (0.94)	1.79 (0.58)	3.83 (0.72)
One-way ANOVA		*F*(5,110.63) = 2.28, *P* = 0.051	*F*(5,576) = 11.53, *P* < 0.001	*F*(5,105.6) = 61.10, *P* < 0.001	*F*(5,576) = 2.65, *P* = 0.022
Hospital working unit					
Critical care units	261 (44.8)	13.10 (2.04)	3.91 (0.94)	3.11 (0.75)	4.12 (1.05)
Inpatient wards	113 (19.4)	12.51 (1.89)	3.55 (0.99)	2.34 (0.93)	4.01 (0.89)
Auxiliary services	26 (4.5)	12.31 (4.20)	3.52 (1.10)	2.40 (1.02)	3.94 (1.11)
Outpatient clinics	163 (28)	12.65 (2.18)	3.87 (0.94)	2.83 (0.84)	4.10 (1.11)
Academic	19 (3.3)	13.05 (1.93)	3.79 (0.87)	2.35 (0.84)	4.01 (0.96)
One-way ANOVA		*F*(4,79.75) = 2.17, *P* = 0.080	*F*(4577) = 3.68, *P* = 0.006	*F*(4,79.50) = 18.83, *P* < 0.001	*F*(4577) = 0.38, *P* = 0.827
Attended Hospital Educational Day Campaign					
No	344 (59.1)	12.71 (2.27)	3.70 (0.92)	2.65 (0.87)	4.07 (0.94)
Yes	238 (40.9)	12.97 (2.10)	3.97 (1.00)	3.10 (0.84)	4.10 (1.16)
*t* test unpaired		*t* = 1.39, df = 580, *P* = 164	*t* = 3.3, df = 580, *P* = 0.001	*t* = 6.03, df = 580, *P* < 0.001	*t* = 0.32, df = 580, *P* = 0.747

### Knowledge and attendance at the Educational Day conference

**Fig. 2.**
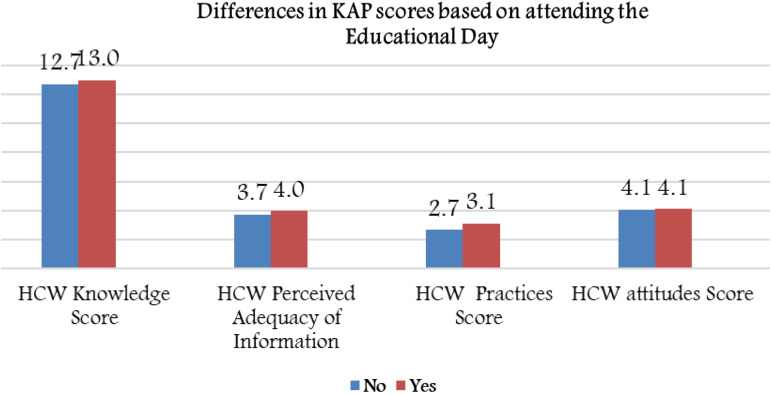
Mean KAP scores and perceived adequacy of information based on attendance at the Educational Day conference.

The mean knowledge score among the whole sample was 12.75 ± 2.2 of a total score of 16. There was no difference in the knowledge scores for clinical role groups, gender or hospital working areas (Table 1). Nearly 40% of the whole sample attended the hospital's Educational Day conference. A higher perceived adequacy of COVID-19 information (mean: 3.97 ± 1.00 *vs.* 3.7 ± 0.92, *P* = 0.001) and better hygienic practices (3.10 ± 0.84 *vs.* 2.65 ± 0.87, *P* < 0.001) were observed among HCWs who attended the Educational Day conference (Fig. 2). However, there was no difference in knowledge scores and attitudes toward selected preventive measures between those who attended and those who did not attend the Educational Day conference.

### KAP score differences based on gender

**Fig. 3.**
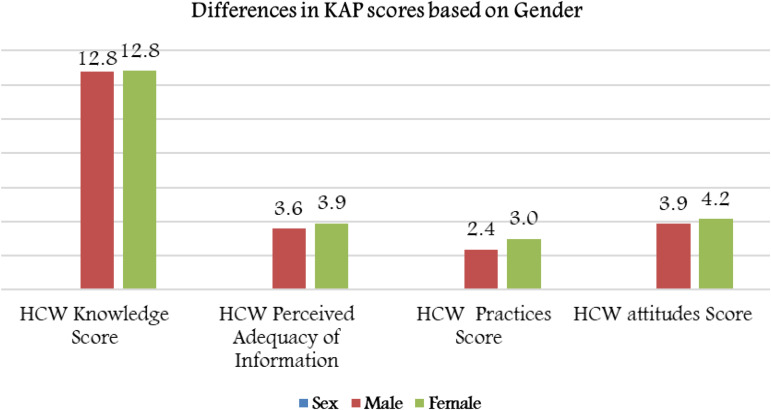
Mean KAP scores and perceived adequacy of information based on gender groups.

The mean hygienic practice score was 2.76 ± 0.91 for the whole sample. There was no difference in knowledge scores between males and females. However, female HCWs scored higher in terms of adherence to hygienic practices (2.98 ± 0.82 *vs.* 2.36 ± 0.90, *P* < 0.001), attitudes toward infection control measures (3.85 ± 0.93 *vs.* 4.15 ± 1.10, *P* = 0.003), and perception of adequacy of knowledge (3.88 ± 0.93 *vs.* 3.58 ± 1.03, *P* = 0.002; Fig. 3).

### Clinical role and clinical area

There was no significant difference in knowledge scores among different HCWs across their different clinical roles. However, nurses had significantly higher hygienic practice scores (3.20 ± 0.68, *P* < 0.001) compared to all levels of physicians, in addition to significantly higher attitude scores toward infection control practices compared to resident physicians (*P* = 0.041).

Taking clinical area assignment into consideration, staff working in critical care units had significantly higher perceived information regarding COVID-19, which was reflected by significant higher hygienic behaviour compliance. The younger HCWs (⩽30 years) had significantly less adherence to hygienic practices compared to older HCWs (31–39 years; *P* < 0.001) but both the young age group and the group working in critical care area did not show any significant difference in terms of attitude toward infection control practices.

### Previous experience with MERS-CoV

**Fig. 4.**
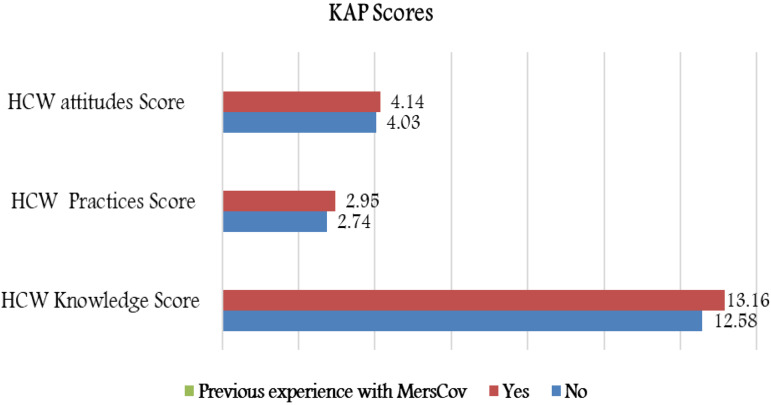
Mean KAP scores based on previous experience with MERS-CoV.

Nearly 40% of our sample had previous experience dealing with confirmed or suspected cases of MERS-CoV. Those who had previous experience with MERS had a significantly higher knowledge scores (13.16 ± 2.02 *vs.* 12.58 ± 2.27, *P* = 0.002) and higher adherence to protective hygienic practices (2.95 ± 0.80 *vs*. 2.74 ±0.92, *P* = 0.003; Fig. 4). However, there was no significant difference in the protective attitude toward domestic hygiene among HCWs who had previous experience with MERS-CoV cases compared with the HCWs who did not.

### Adherence to seasonal influenza vaccination

**Fig. 5.**
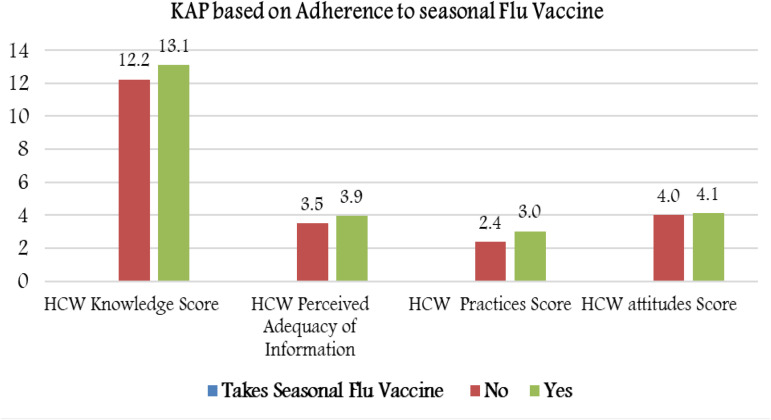
Mean KAP scores based on adherence to seasonal flu vaccinations.

Almost one-third (29.4%) of HCWs did not adhere to flu vaccinations. KAP scores analysed based on adherence to influenza vaccinations showed higher knowledge mean scores (13.00 ± 2.10 *vs.* 12.23 ± 2.25, *P* < 0.001) and hygienic practice scores (3.01 ± 0.81 *vs.* 2.38 ± 0.89, *P* < 0.001; Fig. 5). There was no difference in attitude scores.

### Correlation between scores of knowledge, attitudes and practices

There was a moderate positive correlation between HCW infection control attitudes and their perceived adequacy of information about COVID-19 (*r* = 0.53, *P* value <0.01) (Table 2). In addition, there was a weak but significant correlation between hygienic practice scores and perceived adequacy of information (*r* = 0.32, *P* < 0.01) and with HCW knowledge scores (*r* = 0.22, *P* < 0.01). These findings suggested that education plays an important role in improving HCW attitudes and practices to prevent COVID-19.

**Table 2.** Correlation between scores for knowledge, attitudes and practices

	Hygienic practices score	COVID-19 knowledge score	Perceived adequacy of COVID-19 information
Hygienic practices score	1		–
HCW knowledge score	0.219**		
HCW perceived adequacy of information	0.316**	0.10*	
HCW attitudes score	0.16**	0.06	0.533**

**Correlation is significant at the 0.01 level (2-tailed).

*Correlation is significant at the 0.05 level (2-tailed).

#### HCW's perceived higher fear/stress from COVID-19 than from previous MERS-CoV outbreaks

The HCWs were asked about their worry level from current COVID-19 compared to their worry level from previous MERS-CoV outbreaks. Multivariate logistic regression analysis of the predictors of the HCW's higher stress from COVID-19 compared to MERS-CoV was performed and the results are displayed in Table 3. HCWs previously exposed to MERS-CoV infected/suspected cases were significantly less likely to have higher stress from the COVID-19 outbreak (odds ratio (OR) = 0.646, *P* = 0.042). We found that HCWs who had attended their hospital Educational Day on COVID-19 were less likely to have stress related to COVID-19 compared to stress related to MERS-CoV (OR = 0.654, *P* = 0.035), accounting for other confounders.

**Table 3.** Multivariate analysis of the HCWs' perceived high fear/stress levels from COVID-19 compared to previous MERS-CoV outbreaks

	*B*	Adjusted OR	95% CI for exp(*B*)		*P*-value
			Lower	Upper	
Sex (female)	111	1.118	0.698	1.788	0.643
Age (years)	−0.030	0.970	0.946	0.996	0.023
Clinical role	−0.036	0.964	0.799	1.164	0.704
Hospital working unit	−0.065	0.937	0.802	1.094	0.410
Previously exposed to MERS-CoV-infected patients = yes	−0.437	0.646	0.424	0.984	0.042
Plans to rescheduled leave/absenteeism = yes	0.058	1.060	0.638	1.761	0.823
Takes annual vaccination = Yes	0.048	1.049	0.663	1.661	0.837
Worry level from contracting COVID-19 oneself^a^	0.659	1.933	1.445	2.586	<0.001
Worry level from contracting COVID-19 and transmitting it to family members^a^	0.088	1.092	0.867	1.376	0.454
Attended hospital awareness campaign = Yes	−0.424	0.654	0.441	0.970	0.035
Hygienic practices score 1–4 Likert-rating scale	0.265	1.303	1.004	1.692	0.046
Anxiety from MERS-CoV^b^	0.015	1.015	0.916	1.125	0.780
Anxiety from seasonal flu^b^	−0.087	0.916	0.837	1.004	0.060
COVID-19 knowledge score	0.026	1.026	0.942	1.118	0.557
Perceived adequacy of COVID-19 information score^a^	−0.158	0.854	0.668	1.090	0.204
HCW attitude score^a^	0.242	1.273	1.021	1.588	0.032

Dependent variable = high stress from COVID-19 compared to MERS-CoV. Model significance: *χ*^2^_(16)_ = 103.8, *P* < 0.001, model goodness-of-fit Hosmer–Lemeshow test *χ*^2^_(8)_ = 7.4, *P* = 0.490, model AUC ROC = 74%.

a 1–5 Likert scale.

b 1–10 rating scale score.

HCW worry levels based on contacting COVID-19 themselves was associated with higher stress from COVID-19 disease compared to MERS-CoV (OR = 1.933 times higher, *P* < 0.001).

HCWs' hygienic practice scores converged significantly on higher odds of having high stress from COVID-19 compared to MERS-CoV (OR = 1.303, *P* = 0.046).

Nonetheless, the HCWs' attitude scores correlated positively and significantly with higher odds of being highly stressed from COVID-19 disease (OR = 1.27, *P* = 0.032), accounting for the other predictors.

### MERS-CoV *vs.* COVID-19 comparison of anxiety, knowledge and practice changes

We compared our current analysis with data from the previous study conducted in the same institution during the MERS-CoV outbreak in 2015 [15]. The resulting data analysis (Table 4) suggested that the HCWs' worry levels for contracting COVID-19 and passing it on to their families and friends were significantly lower than those measured during the MERS-CoV (*P* < 0.001). Also, the HCWs' worry levels for contracting COVID-19 was significantly lower than those measured during the previous MERS-CoV outbreak in 2015 (*P* < 0.001). This might be due to in part because this study was done before WHO declared COVID-19 a global pandemic. Interestingly, the proportion of HCWs who underwent annual influenza vaccination at the current time significantly exceeded those measured during the 2015 MERS-CoV outbreak; the difference in percentages of HCWs who immunised annually indicated an 18.86% rise in the proportion of HCWs who immunised against seasonal influenza during this current point of time in 2020 compared to the same period in 2015 (*P* < 0.001). However, comparing the HCWs' perceived hygienic changes and intents to be absent from their work did not differ significantly between the current study and the previous MERS-CoV (2015) study (*P* > 0.05 for each).

**Table 4.** Bivariate comparison on HCW's main perceived concepts between two studies conducted within the same hospital during current (COVID-19) and previous (MERS-CoV) global outbreak times^a^

Measured concepts	MERS-CoV 2015 *N* = 591	COVID-19 *N* = 582	Test statistic	*P*-value
Anxiety (concern of transmitting the viral infection to family)^b^	3.24 (1.20)	2.71 (1.22)	*t*(1069) = 7.24	<0.001
Anxiety (concern of contracting the viral infection)^b^	3.10 (1.04)	2.57 (1.071)	*t*(1069) = 8.3	<0.001
Took annual flu immunisation, *n* (%)	267 (51.74%)	411 (70.6%)	*Z* = 6.42	<0.001
Knowledge score of the specific viral outbreak (%)	68.22% (15.1%) out of 100%	79.7% (13.7%) out of 100%	*t*(1069) = 13.67	<0.001
Behavioural practice changes^c^	2.85 (1.01)	2.82 (0.88)	*t*(1096) = 0.53	0.599
Absenteeism intentions-leave rescheduling, *n* (%)	80 (15.5%)	89 (15.3)	*Z* = 0.097	0.920

a Data from 2015 during the MERS-CoV outbreak as compared to data from early 2020 during the COVID-19-crisis from same HCW population.

b 1–5 rating scale.

c 1–4 Likert rating scale.

## Discussion

The world has experienced several epidemics with novel coronaviruses; namely, SARS-CoV-1, which emerged in China in 2003 followed by Middle East respiratory syndrome coronavirus (MERS-CoV) in the Middle East in 2012, and the current Severe Acute Respiratory Syndrome Corona Virus-2 (SARS-CoV-2) pandemic [16]. MERS-CoV continues to be endemic in Saudi Arabia with weekly reported cases. With the ongoing circulation of MERS-CoV and continuing zoonotic spillover with 70% of the cases resulting from hospital outbreak, the emergence of COVID-19 within the same setting will be overwhelming to healthcare facilities and workers [17]. Therefore, it is of great importance to know the impact of such epidemics on HCWs. This is an expected finding since there are established guidelines on the treatment of MERS-CoV and seasonal influenza and lack of comprehensive knowledge and experience with SARS-CoV-2.

As the understanding of the epidemiology of SARS-CoV-2 evolved, human-to-human transmission was confirmed with the potential for asymptomatic transmission as well [18]. SARS-CoV-2 has also demonstrated a very rapid transmission rate with a reported *R*_0_ = 2.5; i.e. each patient can spread the virus to two other patients [20]. Hospital transmission was also reported and was estimated to account for 41.3% of cases [19]. This highlights the importance of strict infection control measures and continuous HCW education and competency, not only to decrease transmission but to limit HCW anxiety, which will result in better compliance, performance and patient care. These HCWs reported that their main concern was the risk of transmitting the infection to their families (2.71/5) or acquiring it themselves (2.57/5) [21].

In response to the global crisis, as part as hospital preparedness [22], King Saud University Medical City (KSUMC) arranged an Educational Day for all hospital staff in an attempt to increase awareness and improve the preparedness of HCWs. A survey was distributed afterward and completed by 582 HCWs working at King Khalid University Hospital at KSUMC to ensure the adequacy of education. The majority (62.3%) of the responding HCWs were nurses, who were approximately half of the attendees (46.2%), and they reported adequacy of the information given regarding symptoms, treatment, prognosis and prevention of COVID-19 disease. We observed a better hygienic practices and higher perceived adequacy of COVID-19 information among those who attended the hospital's Educational Day conference. However, only 40% of the whole sample attended this activity. Therefore, a different approach is required to improve the attendance of educational activities. Suggested approaches include making the COVID-19 educational activities periodic, making the attendance mandatory for all staff and utilising alternative or additional online modules.

These results showed that a greater proportion (53.8%) still did not get a proper education and, hence, there was a need for more awareness activities and campaigns, including for example but not limited to lectures, departmental educational activity, concise educational leaflets, in addition to newly innovated methods including virtual lectures, smart phone applications (Apps) and hotline access during the current pandemic to answer questions and queries.

COVID-19 pandemic has been accompanied by an overabundance of information that makes it difficult to obtain what is true from false, as most individuals get their information from social media [17], and this might contribute to higher levels of misconception or anxiety. In this survey, the top source accessed by HCWs was hospital announcements (77.8%), which were similar to the main sources used in the previous MERS-CoV outbreak [15]. This finding again highlights the importance of having a dedicated team to provide accurate information from trusted sources as most HCWs rely on it. Social network news remained a source of information among 61.7% of the HCWs, which might elevate anxiety and should be discouraged in future awareness campaigns.

The current study showed that HCWs who had previous experience with MERS had higher knowledge scores and more adherence to protective hygienic practices. These results could be explained by the fact that previous hospital educational campaigns and managing previous MERS-CoV cases may have enhanced their knowledge and intentions to be in compliance with infection control practices [23]. Another speculation is that the occurrence of MERS in Saudi Arabia is ongoing and there is more awareness about it among HCWs [24, 25].

Similarly, HCWs who were more adherent to receiving the annual influenza vaccine had higher knowledge mean score and higher compliance with hygienic practices. The association between the effect of knowledge, among other multimodal interventions, and compliance with influenza vaccination, has been demonstrated in previous studies, including those conducted in Saudi Arabia [26–28]. Influenza vaccine utilisation may indicate general awareness and initiative for self-healthcare.

Most HCWs had strong perceptions of the importance of change in hygienic behaviours, including compliance to hand hygiene, infection control measures, avoiding contact with people who have flu-like symptoms, and decreasing handshaking and social visits. The level of knowledge of HCWs toward viral infection outbreaks during the current COVID-19 pandemic are much higher compared to the previous study conducted in the same institution during MERS-CoV a few years ago [15]. This finding is promising as it is expected that HCWs would be more compliant with infection control measures. For different subgroups we observed higher scores in hygiene practices for HCW attending Educational Day conference, especially among females and nurses. This is directly reflective on the higher number of nurses attending such activities and the predominance of female nurses in our institution.

HCW contribution to proper management of COVID-19 infected patients is substantial, as the more knowledgeable they become, the more likely their management to be more appropriate.

HCW not wearing masks have a 30% chance of developing infection [29], meaning at least one in every three HCW not adherent to PPE will get infected. This highlights the importance of sending correct information to enhance their knowledge which would reflect on their attitudes and practices.

As of 17 August 2020 within the Eastern Mediterranean region, the Kingdom of Saudi Arabia ranks second only to the Islamic Republic of Iran in total number of infected and active cases, follow by Pakistan then Iraq, yet the case fatality rate (CFR) is 1.1 in Saudi Arabia compare to 5.75, 2.14 and 3.31 in Iran, Pakistan and Iraq, respectively [30]. This lower CFR in comparison to high attack rate can be attributed to the healthcare system in Saudi Arabia that can be anticipated in a country that dealt with several outbreaks of Middle East respiratory syndrome coronavirus (MERS-CoV). Moreover, the strict social distancing and lockdown strategies that were implemented in Saudi Arabia since the early stages of the pandemic contributed in slowing down the spread of COVID-19 and decreased the CFR via preventing the healthcare system from overwhelming COVID-19 patients, and thus allowing better and lifesaving care. Evaluations of the related COVID-19 CFR have suggested that CFR was dependent on the efficacy of local response efforts [31, 32]. A study from China, for example, found that the timely supply of adequate medical resources lowered the CFR from around 4.5–0.5% [33]. We suggest that high CFRs in the above-mentioned countries were in part determined by hospital and healthcare surge capacity being exceeded and, as a result, patients potentially receiving suboptimal care during the crisis.

Recently, Nguyen *et al*. reported that front-line HCWs had at least a threefold increased risk of COVID-19 infections [34]. Given the above-mentioned initial high attack rates in Saudi Arabia and low CFR, future research that defines the associated risks and outcomes for HCWs who contracted COVID-19 in Saudi Arabia is advised.

There are several limitations to this study. First, it was done in a single centre and therefore it cannot be generalised to the entire population and this also contributes to selection bias. Second, a self-administered electronic questionnaire was used, which increases the chances of recall bias; however, this was balanced with previous data that was collected using the same method from the same cohort. Third, although there were statically significant differences in knowledge, attitude and practice scores between different groups, but a small difference should be interpreted with caution.

Finally, the study was done early in the pandemic and not much information was available and thus it corresponded to a variable level of anxiety and stress. Despite these limitations, the study has highlighted the importance of addressing HCW stress levels and ensuring providing information from trustable sources, which will all contribute to better compliance with infection control measures and limiting disease spread.

## Conclusion

The HCWs' worry levels regarding contracting or transmitting MERS-CoV were higher than for COVID-19 during the early stage of the COVID-19 pandemic. The current study showed that previous experience with MERS-CoV and subsequent awareness campaigns that were conducted were associated with increased knowledge, adherence to protective hygienic practices and reduction of anxiety toward the COVID-19 pandemic.

## Data Availability

The datasets generated during and/or analysed during the current study are available from the corresponding author on reasonable request.

## Supplementary material

For supplementary material accompanying this paper visit http://dx.doi.org/10.1017/S0950268820001958. click here to view supplementary material
