# Supplementary material for: Knowledge, attitudes and practices of healthcare workers during the early COVID-19 pandemic in a main, academic tertiary care centre in Saudi Arabia
Source: Epidemiol Infect. 2020 Aug 28;148:e203. doi: 10.1017/S0950268820001958 (PMC7492582; doi:10.1017/S0950268820001958)
Supplement: Supplementary file 1 [file S0950268820001958sup001.doc]

**Supplementary Tables S1-S4:**

**Table S1:** Descriptive statistics on healthcare worker indicators of knowledge of COVID-19 disease.

|  | **Incorrect,**  **n (%)** | **Correct,**  **n (%)** |
| --- | --- | --- |
| It can be transmitted by droplets from the patient's coughing or sneezing | 13 (2.2%) | 569 (97.8%) |
| It can be transmitted by contact with patient's tools then touching the nose or eyes | 57 (9.8%) | 525 (90.2%) |
| *The 2019-NCoV can be transmitted from packages shipped from China* | 395 (67.9%) | 187 (32.1%) |
| It can cause severe respiratory symptoms | 13 (2.2%) | 569 (97.8%) |
| It can cause severe neurological symptoms | 361 (62%) | 221 (38%) |
| It may affect the Medical Staff | 13 (2.2%) | 569 (97.8%) |
| There is no vaccine | 38 (6.5%) | 544 (93.5%) |
| There is no specific treatment | 37 (6.4%) | 545 (93.6%) |

**Table S2: Descriptive statistics on healthcare workers’ perceived adequacy of knowledge about COVID-19.**

|  | **Mean (SD)** | **SD*** | **D** | **N** | **A** | **SA** |
| --- | --- | --- | --- | --- | --- | --- |
| I have received sufficient information about NCOV symptoms | 3.94 (1.10) | **33 (5.7%)** | **44**  **(7.6)** | **42 (7.2%)** | 269 (46.2%) | 194 (33.3%) |
| I have received sufficient information about NCOV prognosis | 3.69 (1.10) | 35  (6%) | 52 (8.9%) | 92 (15.8%) | 285 (49%) | 118 (20.3%) |
| I have received sufficient information about NCOV treatment | 3.53 (1.15) | 43 (7.4%) | 68 (11.7%) | 120 (20.6%) | 238 (40.9%) | 113 (19.4%) |
| I have received sufficient information about NCOV transmission routes | 3.90 (1.04) | 31 (5.3%) | 39 (6.7%) | 42 (7.2%) | 313 (53.8%) | 157 (27%) |
| I have received sufficient information about NCOV Prevention | 3.98 (1.10) | 32 (5.5%) | 30 (5.2%) | 53 (9.1%) | 272 (46.7%) | 195 (33.5%) |
| ****SD=Strongly disagree, D=Disagree, N=neither agree/disagree, A=Agree, SA=strongly agree, **p-value of chi-squared goodness-of-fit test.*** | | | | | | |

**Table S3:** Descriptive statistics on healthcare workers’ hygienic practice changes

|  | **Mean (SD)** | **SD*** | **D** | **A** | **SA** |
| --- | --- | --- | --- | --- | --- |
| There is increase in your compliance with hand hygiene at the hospital | 3.22 (1) | **62**  **(10.7%)** | **57**  **(9.8%)** | 155 (26.6%) | *308 (52.9%)* |
| There is increase in your compliance with Universal Precautions (examples: masks & gloves) | 3.28 (0.99) | 61  (10.5%) | 43  (7.4%) | 158 (27.1%) | *320 (55%)* |
| There is increase in your habits of purchasing / obtaining hand sanitizer (example: pocket alcohol gel) | 2.93 (1.14) | 111  (19.1%) | 64  (11%) | 163 (28%) | *244 (41.9%)* |
| There is increase in your avoidance of contact with people having flu symptoms | 3.13 (1.02) | 65  (11.2%) | 72  (12.4%) | 167 (28.7%) | *278 (47.8%)* |
| There is decrease in your social visits (example: visiting friends) | 2.36 (1.17) | 197  (33.8%) | 109 (18.7%) | 146 (25.1%) | 130 (22.3%) |
| There is decrease in your handshaking habits | 2.40 (1.16) | 183  (31.4%) | 116 (19.9%) | 148 (25.4%) | 135 (23.2%) |
| There is decrease in your use of public facilities (example: toilets) | 2.47 (1.17) | 174  (29.9%) | 114 (19.6%) | 143 (24.6%) | *151 (25.9%)* |
| ****SD=Strongly disagree, D=Disagree, A=Agree, SA=strongly agree, **p-value of chi-squared goodness-of-fit test.*** | | | | | |

**Table S4:** Descriptive statistics on healthcare workers’ attitudes toward infection control measures.

|  | **Mean (SD)** | **SD*** | **D** | **N** | **A** | **SA** |
| --- | --- | --- | --- | --- | --- | --- |
| It is important to wear face masks in crowded places during influenza season for flu prevention | 3.83 (1.28) | 53 (9.1%) | 48 (8.2%) | 75 (12.9%) | 175 (30.1%) | 231 (39.7%) |
| It is important to maintain good indoor ventilation during flu season to prevent disease spread | 4.10 (1.16) | 47 (8.1%) | 15 (2.6%) | 43 (7.4%) | 224 (38.5%) | 253 (43.5%) |
| It is important to avoid going to crowded places during flu season | 3.97 (1.22) | 50 (8.6%) | 30 (5.2%) | 54 (9.3 %) | 201 (34.5%) | 247 (42.4%) |
| Hand hygiene is very important to protect from Corona infection | 4.44 (1.15) | 49 (8.4%) | 3 (0.5%) | 6 (1%) | 109 (18.7%) | 415 (71.3%) |
| *SD=Strongly disagree, D=Disagree, N=neither agree/disagree, A=Agree, SA=strongly agree, **p-value of chi-squared goodness-of-fit test. | | | | | | |
